# Early Discharge of Very Preterm Infants Is Not Associated with Impaired Growth up to Three Months Postmenstrual Age: A Prospective Cohort Study

**DOI:** 10.3390/nu17213431

**Published:** 2025-10-31

**Authors:** Rahel Schuler, Vanessa Bethke, Viola Schmidt, Tina Frodermann, Annesuse Schmidt, Martin Wald, Andreas Hahn, Walter A. Mihatsch

**Affiliations:** 1 Department of General Pediatrics and Neonatology, Justus-Liebig-University, Feulgenstrasse 12, D-35392 Giessen, Germany; 2 Divisions of Neonatology, Department of Pediatrics and Adolescent Medicine, Paracelsus Medical University, 5020 Salzburg, Austria; 3 Department of Pediatric Neurology, Justus-Liebig-University, Feulgenstrasse 12, D-35392 Giessen, Germany; 4 Division of Neonatology and Pediatric Intensive Care Medicine, Department of Pediatrics and Adolescent Medicine, University Medical Center Ulm, Eythstr. 24, D-89075 Ulm, Germany; 5 Department of Pediatrics, Balingen Pediatric Hospital, Tuebinger Str. 31, D-72336 Balingen, Germany

**Keywords:** preterm, early discharge, post-discharge growth, family-centered care

## Abstract

**Background/Objectives:** Postnatal growth restriction and duration of hospital stay have been identified as risk factors for adverse neurodevelopment in preterm infants. Implementation of a family-centered care (FCC) program in our institution reduced length of stay in preterm infants. This study evaluates the effect of more early discharge on growth up to three months postmenstrual age (PMA). **Methods:** We conducted a prospective, single-center cohort study in a German level III neonatal unit (October 2020–November 2023) including six consecutive cohorts (n = 184) with progressive FCC implementation. This secondary analysis examined growth at discharge, term-equivalent age (TEA), and three months PMA. **Results:** PMA at discharge significantly decreased from the baseline to intervention cohort 5 (37.8 ± 2.1 vs. 35.7 ± 0.91 weeks PMA; *p* = 0.03). Compared to the baseline cohort, infants in intervention cohort 5 had significantly lower weight, length, and head circumference at discharge. However corresponding Z-Scores did not differ significantly between the cohorts. No significant differences in growth outcomes were observed at TEA or at three months PMA. Furthermore, there were no significant differences in the change in Z-Score for weight, length, and HC from birth to three months PMA. **Conclusions:** Early discharge under FCC did not impair growth to three months PMA, suggesting that early discharge is a safe practice with respect to growth outcomes in preterm infants. Further randomized multicenter studies are needed to confirm these results.

## 1. Introduction

Prolonged duration of hospital stay has been identified as a risk factor for adverse neurodevelopment in preterm infants. Increased parental involvement, especially as primary caregiver for their infant has the potential to shorten hospital stay [1] and enable discharge at a lower gestational age [2]. Therefore, early discharge programs have been established in various countries [3,4]. Mostly, these include discharge with tube feeding and a follow-up program with either neonatal homecare, telemedicine, or outpatient follow-up [3,5]. Several benefits of early discharge on both the infant and parents have been shown. Firstly, early discharge was associated with less moderate to severe neurological impairment at 18–24 months corrected age [4,6]. Secondly, improved breastfeeding outcomes were achieved in some, but not all studies [7,8,9], while none showed a negative effect. Thirdly, mothers in the early discharge group were less anxious at discharge [8] and parental satisfaction with early discharge was high [2,10]. Finally, unplanned readmissions did not increase [2,4].

Postnatal growth restriction is common in preterm infants [11,12]. The association of preterm infant growth and neurodevelopment has been addressed by several observational studies and nutritional intervention studies [13].

Several cohort studies have examined the relationship between early growth and later neurodevelopment in preterm infants. Franz et al. (n = 219, <30 weeks PMA, <1500 g) found that early weight gain predicted outcomes at 5 years, with post-discharge head circumference (HC) growth linked to cognitive performance [14]. Similarly, Belfort et al. (n = 613) reported modest associations between early weight/HC gain and better cognitive and motor outcomes at 18 months [15]. Other studies also identified post-discharge HC growth as a key predictor of later cognitive function [16,17]. In contrast, one study (n = 123) found length growth—not weight or HC—to be most strongly associated with neurodevelopment at age 3 [18]. However, a large multicenter study found no association between growth of extremely preterm infants during the first two years of life and neurodevelopmental outcomes at 10 and 15 years of age [19].

For infants discharged early, adequate growth and prevention of growth faltering is recommended at least until term-equivalent age (TEA). Studies investigating weight gain following early discharge up to TEA are sparse and found no significant difference compared to infants discharged according to standard protocols [7,20]. To our knowledge, no studies so far have comprehensively analyzed the effect of early discharge on weight, length, and head circumference (HC) beyond TEA. Therefore, the aim of the present study was to assess the growth outcome of very low birth weight preterm infants up to three months postmenstrual age (PMA) after early discharge, to exclude that post-discharge growth faltering counterbalances the positive effects of early discharge.

## 2. Materials and Methods

Study design: This study is part of a prospective longitudinal study performed in a German perinatal level III center assessing the impact of enhancement of family-centered care (FCC). It is registered at clinicaltrials.gov (NCT05286983) and received approval from the local Institutional Review Board prior to initiation (AZ 153/20) [21].

The primary outcome of the overall study is PMA at discharge. In a previous analysis, we demonstrated a significantly lower PMA at discharge following the FCC intervention.

The present analysis examines infant growth trajectories until three months PMA, representing one of the study’s predefined secondary outcomes.

Inborn and outborn preterm infants of a PMA of ≤32 + 0 weeks and/or birth weight ≤1500 g were considered eligible. Infants were ineligible if they had severe congenital anomalies (e.g., cyanotic heart disease), if decision for palliative care was taken before study entry, or if parents suffered from a severe active psychiatric disease (e.g., psychosis). Written informed consent was obtained from the parents prior to inclusion.

Neonatal morbidities were defined as follows: bronchopulmonary dysplasia (BPD) according to the physiological definition by Walsh [22], intraventricular hemorrhage (IVH) > grade III and periventricular leukomalacia (PVL) were diagnosed by ultrasound, retinopathy of prematurity (ROP) > Stage 3 or treatment of ROP and necrotizing enterocolitis (NEC) > Stage 2.

The baseline cohort (October 2020–May 2021) included 45 infants prior to enhanced FCC implementation. The following five intervention cohorts consisted of infants recruited consecutively within 6-month-periods from June 2021 till November 2023. The enhanced FCC intervention included multiple components designed to empower parents to assume the role of primary caregiver. The specific elements of the FCC intervention were implemented stepwise [2].

In the baseline cohort, infants were typically discharged upon completion of full oral feeding. As part of the FCC intervention, biweekly staff workshops were held to emphasize the role of the medical team in empowering parents to become the primary caregivers of their infants. This included gradually transferring caregiving responsibilities such as tube feeding to the parents. A key component of the FCC intervention was therefore the structured and early training of parents in nasogastric tube feeding during the neonatal intensive care unit (NICU) stay.

As a result, earlier discharge with continued home tube feeding became standard practice. Neonatal homecare after discharge was offered to all parents throughout the study and was strongly recommended, but not mandatory for early discharge with tube feeding. Participating families were visited at home once a week for 12 weeks after discharge.

According to the feeding protocol of the neonatal unit, fortification of breastmilk was initiated after complete meconium passage and was continued at least until TEA, irrespective of the time of discharge. If the weight and head circumference were above the 10th percentile (Fenton growth chart) at TEA [23], fortification was discontinued. Infants who were breastfed received the fortification before breastfeeding diluted in a small amount of pumped breastmilk. Infants whose mothers did not provide sufficient breastmilk received term formula with breastmilk fortifier. The feeding protocol remained unchanged throughout the study.

Anthropometric data (weight, body length, and head circumference) were measured routinely during the hospital stay and outpatient follow-up. The largest possible fronto- occipital circumference of the head and body length were measured with a flexible, but non-extensible tape. Length was measured with the crown-heel technique. The present study evaluated growth outcomes following early discharge at discharge, at TEA, and at three months PMA. Changes in Z-Scores for weight, length, and HC from birth to three months PMA were also assessed. For 22 and 23 weeks PMA, the Voigt growth charts were used [24]. From 24 weeks PMA up to TEA, Fenton growth charts were used [23]. At 3-months PMA World Health Organisation growth standards were used for weight, length, and HC [25].

Data Collection: Patient characteristics were extracted from the inpatient and outpatient medical records and from the medical records of the neonatal homecare program.

Statistical analysis: Data analysis was conducted using SPSS version 28.9.1.1 [14]. (IBM Corp., Armonk, New York, NY, USA). Data were presented as the mean (standard deviation, SD) or as number (percentage) as appropriate. The chi-square test was used to compare categorical variables between the five cohorts (Table 1). ANOVA was used to compare continuous variables between the groups and, in case of a statistically significant difference between the groups, post hoc Dunnett test comparing cohort 5 with the baseline cohort was used (Table 1 and Table 2). A *p*-value < 0.05 was considered statistically significant.

## 3. Results

Of 260 principally eligible infants, 76 (29.3%) had to be excluded. A total of 13 parents declined consent, 23 were not approached due to staffing shortage, 34 fulfilled exclusion criteria, and 6 infants died during hospital stay and were excluded from further analysis. No infant died after discharge up to three months PMA.

Clinical data and complications during hospital stay of all 184 surviving infants in the six cohorts are summarized in Table 1. There were no statistically significant differences observed regarding PMA at birth, mode of delivery, sex, anthropometric data at birth, neonatal morbidities and multiples; significant differences between the single cohorts were found for in-hospital mortality.

Table 2 depicts discharge, growth, and tube feeding outcomes. Infants in intervention cohort 5 were discharged at a significantly lower PMA than infants in the baseline cohort (37.8 (±2.1) vs. 35.7 (±0.91) weeks; *p* = 0.03). Compared to the baseline cohort, infants in intervention cohort 5 had significantly lower weight, length, and head circumference at discharge. However, Z-Scores did not differ significantly between the cohorts. No significant differences were observed in any of the other growth outcomes. There was no significant difference in the change in Z-Score from birth to 3 months PMA across all cohorts for weight, length, and head circumference.

Significantly more infants were discharged with tube feeding (*p* < 0.01) in intervention cohort 5. There were significant differences in PMA at removal of the tube (*p* = 0.01) and the duration of tube feeding after discharge (*p* = 0.01).

## 4. Discussion

This is the first comprehensive analysis of the effect of early discharge within an FCC framework on growth outcome up to three months PMA in very low birth weight infants. At three months PMA, no differences were observed in age-adjusted Z-Scores for weight, length, and HC. Furthermore, across all cohorts, changes in Z-Scores for these parameters from birth to three months PMA did not differ significantly. Although this is a single-center study, these findings suggest that significantly earlier discharge of very preterm infants does not impair postnatal growth up to three months PMA.

Due to earlier discharge at a lower gestational age, weight, length, and head circumference at discharge were reduced in intervention cohort 5; however, Z-Scores remained unaffected.

No data is available from the literature regarding length and head circumference after early discharge, and only a few studies have analyzed weight up to TEA [7,20,26]. Ahnfeldt et al. reported on a cohort of 900 preterm infants born at 32–34 weeks PMA and found no significant difference in weight-for-age Z-Scores at final discharge (approximately at TEA) between infants in the early versus standard discharge group [7]. Similarly, Holm et al. observed no significant differences in median weight-for-age Z-Scores near TEA among infants born at a median gestational age of 30 and 34 weeks PMA, respectively [26]. In line with these findings, Örtenstrand et al. reported similar daily weight gain up to TEA in a cohort of 90 preterm infants born at 31 weeks PMA, regardless of discharge timing [20]. These findings are consistent with our results, which demonstrated no differences in age-adjusted Z-Scores for weight, length, and head circumference at TEA, despite a significantly earlier discharge of more immature preterm infants born at 28–29 weeks PMA. Only one other early discharge study of 123 preterm infants mentioned growth up to three months PMA after early discharge and did not find a difference between the early discharge group and the standard group, while growth was not specified further [27]. Collectively, the available evidence from the literature and our data support the appropriateness of early discharge in preterm infants, as it does not appear to adversely affect post-discharge growth.

Although the optimal post-discharge nutritional and growth trajectories for preterm infants remain to be fully defined [28], the available evidence supports the importance of ensuring adequate growth at least until TEA, and likely through the early post-discharge period, to support favorable neurodevelopmental outcomes [14] without increasing the risk of later metabolic disease [29].

Our findings indicate that early discharge is safe and does not negatively impact growth up to three months PMA, a period that possibly represents a critical window for neurodevelopment.

PMA at removal of the tube was delayed for infants after early discharge within the framework of FCC. In intervention cohort 5, a significantly higher percentage of infants was discharged with tube feeding. Delayed removal of the tube is commonly observed following early discharge [7,26]. In many NICUs, tube removal is a prerequisite for discharge [3,30], which may lead to a pronounced focus on facilitating discharge through early tube removal. If the infant is already at home, the removal of the tube becomes less important. In two other early discharge studies, the PMA at the time of tube removal was reported as 37 and 38 weeks PMA, which is similar to our cohorts [10,31]. For the majority of infants, tube feeding was necessary for approximately 2 weeks post-discharge, which is slightly longer than 8 and 9 days reported in two studies involving more mature preterm infants [10,27]. Given that the duration required to achieve full oral feeding is negatively correlated with PMA, this finding is not unexpected [32].

This study has several limitations. First, it was a single-center study. Therefore, the results cannot easily be transferred to other neonatal units. Second, this was not a randomized trial, and potential confounding factors related to secular trends over the study period cannot be excluded. Factors such as the impact of COVID-19 or staffing changes may have influenced outcomes, independently of the FCC intervention. Third, the overall number of preterm births declined during the study time, resulting in smaller cohort sizes than expected and therefore limiting the generalizability of the findings. Fourth, there is missing growth data after discharge due to loss of follow-up. Anthropometric data at TEA was only available in 80% of the infants because no routine outpatient appointment was scheduled at this time point. However, at three months PMA, 91% of infants could be analyzed (23 of 184 infants were lost to follow-up). We purposely refrained from data imputation of any kind since this may introduce additional bias and would rely on strong statistical assumptions, which are not verifiable. Fifth, adherence to the recommended continuation of breast milk fortification after discharge was not assessed, although guidance was provided to parents by the neonatal homecare team. Sixth, the time point of removal of the tube was only assessed from intervention cohort 3 once discharge with tube feeding became standard practice.

## 5. Conclusions

In conclusion, early discharge of very low birth weight preterm infants with ongoing tube feeding did not compromise growth, as evidenced by comparable Z-Scores for weight, length, and head circumference at TEA and at three months PMA. These findings support the notion that early discharge does not adversely affect postnatal growth in preterm infants. With appropriate neonatal homecare support, parents were able to successfully manage care at home, while their infants maintained adequate growth trajectories following discharge, thereby excluding that growth restriction counters the positive effects of early discharge. These findings should be confirmed in a multicenter study, ideally using a randomized design, to strengthen the evidence and assess generalizability.

## Figures and Tables

**Table 1:** Patient characteristics of all cohorts during hospital stay.

	Baseline Cohort	Cohort 1	Cohort 2	Cohort 3	Cohort 4	Cohort 5	ANOVA or Chi Square Test as Appropriate *p* Value
Discharged alive, n	48	35	26	27	30	18	
In hospital death, n	3	0	0	0	0	3	<0.01
PMA at birth, weeks (SD)	28.5 (±2.9)	29.2 (±2.7)	27.9 (±2.8)	29.3 (±2.8)	28.1 (±2.9)	29.1 (±2.2)	0.41
Cesarean section n (%)	43 (89.6%)	35 (100.0%)	26 (100.0%)	26 (96.3%)	29 (96.7%)	18 (100%)	0.11
Male sex, n (%)	18 (37.5%)	18 (51.4%)	15 (57.7%)	12 (44.4%)	16 (53.3%)	11 (61.1%)	0.43
Multiples, n (%)	17 (35.4%)	6 (17.1%)	8 (30.8%)	10 (37.0%)	9 (30%)	2 (11.1%)	0.21
BPD, n (%)	6 (12.5%)	5 (14.3%)	4 (15.4%)	2 (7.4%)	0 (0%)	0 (0%)	0.17
IVH ≥ III, n (%)	1 (2.1%)	0 (0.0%)	1 (3.8%)	1 (3.7%)	2 (6.7%)	1 (5.6%)	0.72
PVL, n (%)	1 (2.1%)	0 (0.0%)	0 (0.0%)	1 (3.7%)	0 (0.0%)	0 (0%)	0.65
ROP ≥ 3, n (%)	3 (6.3%)	2 (5.7%)	1 (3.8%)	0 (0.0%)	0 (0.0%)	1 (6.3%)	0.6
NEC ≥ 2, n (%)	0 (0.0%)	1 (2.9%)	0 (0.0%)	0 (0.0%)	1 (3.3%)	1 (5.6%)	0.51
FIP, n (%)	3 (6.3%)	1 (2.9%)	0 (0.0%)	0 (0.0%)	0 (0.0%)	0 (0%)	0.3

PMA = postmenstrual age, BPD = bronchopulmonary dysplasia, IVH = intraventricular hemorrhage, PVL = periventricular leukomalacia, ROP = retinopathy of prematurity, NEC = necrotizing enterocolitis, FIP = focal intestinal perforation, data given as mean, SD = standard deviation.

**Table 2:** Discharge, growth, and tube feeding outcome of all cohorts until 3 months PMA.

	Baseline Cohort	Cohort 1	Cohort 2	Cohort 3	Cohort 4	Cohort 5	ANOVA *p* Value
discharged alive, n	48	35	26	27	30	18	
PMA at discharge, weeks (SD)	37.8 (±2.1)	37.5 (±2.9)	37.8 (±4.0)	36.9 (±2.8)	36.4 (±1.9)	35.7 ^#^ (±0.91)	0.03
Neonatal homecare	38 (79.2%)	22 (62.9%)	21 (80.8%)	19 (70.4%)	25 (83.3%)	15 (83.3%)	0.33
Tube feeding at discharge, n (%)	3 (6.3%)	4 (11.4%)	12 (46.2%)	13 (48.1%)	22 (73.3%)	12 ^#^ (66.7%)	<0.01
PMA at removal of tube, weeks (SD)	35.9 (±1.9)	35.9 (±2.3)	37.0 (±2.1)	36.8 (±1.9)	37.6 (±2.6)	36.2 (±2.5)	0.01
Removal of tube after discharge, days (SD)			10.0 (±7.5)	13.5 (±7.9)	13.6 (±8.0)	11.8 (±15.9)	0.77
Follow-up at TEA	39 (81%)	25 (71%)	23 (88%)	21 (78%)	26 87%)	13 (72%)	0.72
Follow-up at 3 months	44 (92%)	32 (91%)	23 (88%)	23 (85%)	29 (97%)	17 (94%)	0.49
Weight, grams (SD)							
Birth	1088 (±394)	1174 (±414)	1006 (±377)	1261 (±386)	1069 (±341)	1193 (±315)	0.15
Z-Score birth	−0.35 (±0.9)	−0.35 (±0.98)	−0.48 (±0.97)	−0.09 (±0.89)	−0.29 (±0.93)	−0.12 (±0.65)	0.64
Discharge	2754 (±477)	2669 (±498)	2624 (±595)	2651 (±601)	2368 (±458)	2275 (±247)	<0.001
Z-Score discharge	−0.63 (±0.65)	−0.65 (±0.85)	−0.72 (±0.59)	−0.46 (±0.79)	−0.9 (±0.94)	−0.8 (±0.69)	0.35
TEA	3156 (±414)	3151 (±654)	3080 (±441)	3315 (±496)	3165 (±613)	3337 (±472)	0.60
Z-Score TEA	−0.78 (±0.86)	−0.57 (±0.98)	−0.79 (±0.89)	−0.08 (±0.63)	−0.55 (±0.80)	−0.53 (±0.69)	0.11
3 months PMA	5697 (±890)	5787 (±902)	5650 (±998)	5847 (±732)	5756 (±725)	5865 (±813)	0.95
Z-Score 3 months PMA	−0.59 (±1.07)	−0.66 (±1.47)	−0.97 (±1.39)	−0.56 (±1.04)	−0.47 (±1.03)	−0.38 (±1.18)	0.68
Length, cm (SD)							
Birth	36.9 (±4.7)	36.7 (±4.8)	35.5 (±5.3)	38.0 (±4.6)	36.1 (±4.0)	37.8 (±4.4)	0.38
Z-Score birth	−0.06 (±1.2)	−0.61 (±1.5)	−0.28 (±1.47)	−0.04 (±1.1)	−0.29 (±1.2)	0.08 (±1.1)	0.33
Discharge	47.7 (±2.3)	46.9 (±2.0)	46.8 (±2.6)	46.9 (±3.1)	45.3 (±3.6)	44.7 (±2.0)	0.02
Z-Score discharge	−0.29 (±0.95)	−0.52 (±1.15)	−0.52 (±1.13)	−0.20 (±1.11)	−0.78 (±1.20)	−0.65 (±0.90)	0.31
TEA	48.7 (±2.0)	46.3 (±3.2)	48.3 (±3.2)	48.7 (±2.4)	48.8 (±3.9)	48.8 (±2.7)	0.99
Z-Score TEA	−0.78 (±0.92)	−0.89 (±1.33)	−0.89 (±1.33)	−0.31 (±1.03)	−0.75 (±1.30)	−1.06 (±1.26)	0.65
3 months PMA	60.1 (±3.0)	59.9 (±3.3)	60.3 (±3.5)	60.4 (±3.0)	60.0 (±2.8)	60.1 (±2.5)	0.88
Z-Score 3 months PMA	0.5 (±1.22)	−0.46 (±1.68)	−0.43 (±1.77)	−0.31 (±1.43)	−0.32 (±1.27)	−0.34 (±1.15)	0.72
Head circumference, cm (SD)							
Birth	25.2 (±3.2)	26.1 (±3.1)	24.3 (±3.4)	26.0 (±3.0)	25.6 (±2.8)	26.5 (±2.3)	0.13
Z-Score birth	−0.49 (±1.0)	−0.38 (±1.1)	−0.80 (±1.3)	−0.58 (±1.1)	−0.21 (±1.3)	−0.09 (±0.9)	0.29
Discharge	33.0 (±1.57)	33.0 (±1.41)	32.5 (±1.30)	32.5 (±1.78)	31.8 (±1.89)	31.3 (±0.90)	<0.001
Z-Score discharge	−0.46 (±0.89)	−0.19 (±0.91)	−0.49 (±1.11)	−0.18 (±0.88)	−0.55 (±1.09)	−0.57 (±0.91)	0.43
TEA	33.6 (±1.15)	34.1 (±1.40)	33.7 (±1.62)	33.9 (±2.29)	34.9 (±2.45)	34.2 (±1.54)	0.26
Z-Score TEA	−0.77 (±0.82)	−0.24 (±1.04)	−0.62 (±1.29)	−0.24 (±1.41)	0.19 (±1.44)	−0.65 (±1.06)	0.12
3 months PMA	40.3 (±1.58)	40.1 (±1.48)	40.0 (±1.73)	40.2 (±1.66)	40.0 (±1.51)	40.0 (±0.98)	0.93
Z-Score 3 months PMA	0.30 (±1.07)	−0.03 (±1.26)	−0.33 (±1.49)	−0.01 (±1.29)	−0.06 (±1.24)	−0.05 (±0.89)	0.52
Change in Z-Score from birth to 3 months PMA							
Weight	−0.72 (±1.08)	−0.17 (±1.11)	−0.45 (±1.02)	−0.51 (±1.10)	−0.16 (±1.09)	−0.24 (±1.28)	0.82
Length	0.01 (±1.46)	0.33 (±1.59)	−0.31 (±1.36)	−0.42 (±1.51)	−0.08 (±1.41)	−0.42 (±1.61)	0.41
Head Circumference	0.67 (±1.07)	0.42 (±1.28)	0.42 (±1.28)	0.60 (±1.33)	0.08 (±1.20)	0.04 (±0.99)	0.31

PMA = postmenstrual age, data given as mean, TEA = term-equivalent age, SD = standard deviation. ^#^ Post hoc Dunnett test, significantly different from baseline cohort (*p* < 0.05).

## Data Availability

The raw data supporting the conclusions of this article will be made available by the authors on request.

## Abbreviations

The following abbreviations are used in this manuscript:

BPD	Bronchopulmonary dysplasia
FCC	Family-centered care
FIP	Focal intestinal perforation
HC	Head circumference
IVH	Intraventricular hemorrhage
NEC	Necrotizing enterocolitis
NICU	Neonatal intensive care unit
PVL	Perivetnricular leukomalacia
PMA	Postmenstrual age
ROP	Retinopathy of prematurity
TEA	Term equivalent age
